# A LysR-Type Transcriptional Regulator, RovM, Senses Nutritional Cues Suggesting that It Is Involved in Metabolic Adaptation of *Yersinia pestis* to the Flea Gut

**DOI:** 10.1371/journal.pone.0137508

**Published:** 2015-09-08

**Authors:** Viveka Vadyvaloo, Angela K. Hinz

**Affiliations:** Paul G. Allen School for Global Animal Health, Washington State University, Pullman, Washington, 99164, United States of America; Quuen's University Belfast, UNITED KINGDOM

## Abstract

*Yersinia pestis* has evolved as a clonal variant of *Yersinia pseudotuberculosis* to cause flea-borne biofilm–mediated transmission of the bubonic plague. The LysR-type transcriptional regulator, RovM, is highly induced only during *Y*. *pestis* infection of the flea host. RovM homologs in other pathogens regulate biofilm formation, nutrient sensing, and virulence; including in *Y*. *pseudotuberculosis*, where RovM represses the major virulence factor, RovA. Here the role that RovM plays during flea infection was investigated using a *Y*. *pestis* KIM6+ strain deleted of *rovM*, Δ*rovM*. The Δ*rovM* mutant strain was not affected in characteristic biofilm gut blockage, growth, or survival during single infection of fleas. Nonetheless, during a co-infection of fleas, the Δ*rovM* mutant exhibited a significant competitive fitness defect relative to the wild type strain. This competitive fitness defect was restored as a fitness advantage relative to the wild type in a Δ*rovM* mutant complemented *in trans* to over-express *rovM*. Consistent with this, *Y*. *pestis* strains, producing elevated transcriptional levels of *rovM*, displayed higher growth rates, and differential ability to form biofilm in response to specific nutrients in comparison to the wild type. In addition, we demonstrated that *rovA* was not repressed by RovM in fleas, but that elevated transcriptional levels of *rovM in vitro* correlated with repression of *rovA* under specific nutritional conditions. Collectively, these findings suggest that RovM likely senses specific nutrient cues in the flea gut environment, and accordingly directs metabolic adaptation to enhance flea gut colonization by *Y*. *pestis*.

## Introduction

The bubonic plague causing bacillus, *Yersinia pestis* can be efficiently transmitted to a mammalian host through the formation of a robust biofilm in the foregut proventriculus of its flea vector. To understand the molecular mechanisms that direct biofilm blockage in the flea, the transcriptional response of *Y*. *pestis* from blocked fleas in comparison to *Y*. *pestis* grown at temperature-matched *in vitro* culture conditions was previously characterized [1]. It was observed that the *rovM* gene encoding a LysR-type transcriptional regulator (LTTR), was highly upregulated in the flea gut only [1].

The LTTRs comprise a large family in prokaryotes and regulate diverse sets of genes involved in virulence, metabolism, motility and quorum sensing [2]. The *Y*. *pestis* RovM protein shares >65% overall identity with homologous LTTRs, LrhA/HexS/PecT/HexA, which regulate biofilm formation, synthesis of adhesins, extracellular polysaccharide, motility, exoenzymes, and exolipids, amongst other essential virulence factors in pathogenic *Escherichia coli*, *Serratia* spp., *Erwinia* spp. and entomopathogens, *Photorhabdus temperata* and *Xenorhabdus nematophila* [3–8]. Unlike the entomopathogens *P*. *temperata* and *X*. *nematophila* however, *Y*. *pestis* is avirulent to fleas [9].

Heroven *et al*. [10] determined that the RovM transcriptional regulator of *Y*. *pseudotuberculosis*, in response to specific environmental nutritional cues, is a key virulence regulator whose elevated protein levels causes hypermotililty and an inability to disseminate to deeper tissues during infection in mice [10]. Importantly, *Y*. *pseudotuberculosis* RovM is able to modulate the activity of *rovA*, that encodes another major virulence transcriptional regulator that indirectly controls cell invasion in gut tissues [10]. The RovM protein was able to repress *rovA* in minimal medium [10], and this was mediated through the global metabolic regulatory protein, CsrA and its associated regulatory non-coding RNAs [11]. Overall, the role of RovA in regulating virulence has been well defined in *Y*. *pseudotuberculosis* [10, 12], *Y*. *enterocolitica* [13, 14] and *Y*. *pestis* [15]. Specifically, in *Y*. *pestis*, RovA has been shown to activate the pH 6 antigen locus (*psaEFABC*), responsible for the synthesis and transport of the PsaA fimbriae that enhance resistance to phagocytosis by macrophages; and both RovA and PsaA are required for full virulence in a mouse model of bubonic plague [15]. Consistent with these findings, upregulation of *rovM* in the flea was accompanied by significant downregulation of *rovA* and *psaE* [1]. Conversely, *rovA* is >5 fold up-regulated while *rovM* is down-regulated during bubonic plague [1].

Given the significance of RovM and its homologs in bacterial pathogenicity, we predicted that that *Y*. *pestis* RovM maintains conserved roles in regulating biofilm formation, repressing *rovA* and/or its mammalian virulence regulon, and/or nutrient sensing to enhance *Y*. *pestis* infection of the flea host. To test this we constructed a *Y*. *pestis* KIM6+ Δ*rovM* mutant and characterized its role, *in vitro*, using assays that assessed its growth, biofilm formation and transcription of both *rovM* and *rovA*, as well as, *in vivo* during flea gut infection to assess its biofilm-mediated gut blockage and survival abilities. Our results indicate that the transcriptional regulator RovM can regulate biofilm in response to specific nutritional cues, e.g. arginine, but does not control *rovA* expression in the flea gut. Significantly, RovM expression appears to be correlated with nutrient sensing and acquisition, and is important for efficient adaptation of *Y*. *pestis* to the flea gut environment.

## Materials and Methods

### Bacterial strains, media and growth conditions

The bacterial strain used in this study was *Y*. *pestis* KIM6+, which lacks the 70-kb virulence plasmid that encodes the T3SS [16]. All growth studies were performed at ~23°C using a Bioscreen C (Growth Curves USA, Piscataway, NJ) shaking incubator, following a prior successive conditioning growth step in the chosen medium used in the study. In this study, besides the rich complex LB medium, two chemically defined media for specific culture of *Y*. *pestis* were used. The one is a complex defined medium called TMH [17] and the other is a minimal medium, which we refer to as Sebbane minimal media (SMM) [18]. Both of the *Y*. *pestis* defined media serve as a base medium to which a novel carbon or nitrogen source can be added. The TMH medium promotes biofilm formation in *Y*. *pestis* and is supplemented with 0.2% galactose and 1*μ*g/mL hemin [19]. TMH media not containing arginine (arg) was also used. To simplify reference to the TMH medium supplemented with 0.2% galactose and 1*μ*g/mL hemin containing or lacking arg, we refer to them as TMH-gal^+^ or TMH-gal^+^arg^-^.

The SMM medium contains the minimal basic metabolites that are necessary for *Y*. *pestis* growth, including the five amino acids for which it is auxotrophic (isoleucine, valine, phenylalanine, methionine and glycine) [18]. For growth studies to determine the effect of the novel nutrient on *Y*. *pestis* growth, the SMM medium, was supplemented with the sugars, glucose, galactose, maltose, arabinose and N,N’-diacetylchitobiose (Sigma) at a concentration of 0.2% (w/v). Alternately, the amino acids, glutamine (gln), arg and histidine (his) that are not normally contained in SMM were supplemented into SMM at concentrations at which they are found in TMH medium, which are 100mM, 24mM and 2mM respectively. The growth studies were performed for 3–4 independent biological replicates. Growth rate was calculated using linear regression analysis of the exponential phase of growth.

### Biofilm assays

To determine ability to form biofilm in LB medium, bacteria were grown in LB broth supplemented with 4 mM CaCl_2_ and 4 mM MgCl_2_ for 18 h at room temperature and diluted to *A*_600nm_ 0.02 in the same medium. To determine ability to form biofilm in TMH-gal^+^ or TMH-gal^+^arg^-^ medium, bacteria were grown in TMH supplemented with 0.2% galactose only for 18h at room temperature. These cultures were then first diluted 1:10 in phosphate-buffered-saline (PBS) and subsequently to 1:400 in either TMH-gal^+^ or TMH-gal^+^arg^-^ medium. Aliquots of 100 μl were added to wells of 96-well polystyrene dishes, and incubated with shaking at 250 rpm for 48h at ambient room temperature. Wells were washed three times with water to remove media and planktonic cells and the adherent biofilm was stained with 200 μl of 0.05% safranin for 20 min. Wells were washed three times with water and plates were left to dry for 48h. The bound safranin dye was solubilized with 200 μl of 30% acetic acid for 30mins and the *A*_450nm_ was measured immediately. The assays were performed using 3–4 independent biological replicates with three technical replicates each.

### *Y*. *pestis* mutagenesis and complementation

The *Y*. *pestis* Δ*rovM* mutant strain was created using the pKOBEG method [20] in which the entire target gene was replaced by a Tn703 kanamycin cassette from pUC4K (Amersham). The primer pairs, lxmarovMf/lxbarovMr and rxbarovMf/rhindrovMr (Table 1) were used to generate the left and right flanking regions of the *rovM* gene from *Y*. *pestis* KIM6+ genomic DNA by PCR. These two PCR fragments were cloned into the XmaI/XbaI and XbaI/HindIII sites of pUC19 respectively. The kanamycin cassette was amplified using the kanxbaf and kanxbar primers (Table 1) and cloned into the XbaI site of the pUC19 plasmid containing the *rovM* flanking regions. The new plasmid pUC19*rovM*500flanking was used as the template to generate the linear PCR fragment using primers lxmarovMf and rhindrovMr, for mutation using the pKOBEG method [20]. Mutations were verified by DNA sequencing. The Δ*rovM*::kan mutant is referred to as just Δ*rovM* here. Primers lxmarovMf and rhindrovMr were used to generate a fragment containing the *rovM* gene and native promoter region from *Y*. *pestis* KIM6+ genomic DNA and this fragment was cloned into a high copy number plasmid TOPO-pCR2.1 (pCR_rovM). The fragment containing the *rovM* gene and native promoter region was then isolated after digesting the pCR_rovM plasmid with BamHI and XhoI and was cloned into the corresponding sites on the low copy number plasmid pACYC177 (pACrovM). Constructs were verified by DNA sequencing. The *ΔrovM* mutant was transformed by electroporation, independently with either pACrovM or pCR_rovM to achieve *in trans* complementation of the *rovM* mutation. The *Y*. *pestis ΔrovM* mutant and wild type strains were transformed with an empty pACYC177 plasmid and a wild type strain was transformed with pCR_rovM construct to use in comparative *rovM* over-expression experiments. All experiments were subsequently performed with the strains harboring plasmids, unless otherwise indicated. All strains and primers used in this study are listed in Table 1.

**Table 1:** Oligonucleotides, plasmids and strains used in this study.

A. Cloning and mutagenesis primers			
**gene target**	**primer**	**sequence (5’– 3’)**	
*rovM*	lxma*rovM*	GTT AGC CCG GGA AAA TCC TGT AAA T (F)	
	lxba*rovM*	CGA GGT CTA GAT TAA TTA TCG GAC G (R)	
	rxba*rovM*	GAT TAA GTA GTC TAG ATT TAA TTC ATC ATC AC (F)	
	rhind*rovM*	ACT AAA GCT TAA TTT ACA AAC ATG C (R)	
kan cassette	kanxba F	GCA GGT CTA GAG GGG AAA GCC ACG TTG TGT C (F)	
	kanxba R	GGG GGT CTA GAC TGA GGT CTG CCT CGT GAA GAA (R)	
B. Taqman primers-probe sets			
**gene target**	**primer**	**sequence (5’– 3’)**	
*Crr*	primers	GCC CTC TGG CAA TAA AAT GG (F); AGC ATG GTT GGT CTC GAA AAT T (R)	
	probe	CTC CTG TTG ACG GCA TCG GT	
*rovM*	primers	CTCAGGCGGGTGTTCCAT (F); GCTCTTACTGCCGCTCGAAT (R)	
	probe	CGTATTGCCTACGTGGCCTCTTCACTTTCT	
*rovA*	primers	GCA CGA TTA GTT CGC GTT TG (F); TTT GAG TCA GTT CCA ACG GTT TC (R)	
	probe	CGC GCA TTA ATT GAC CAT CGG	
C. plasmids			
**Plasmid**	**description**	**source**	**encoded antibiotic resistance**
pUC4K	Contains Tn703 Kanamycin casette, pUC ori	Amersham Biosciences	carbenicillin, kanamycin
pACYC177	p15A ori, low copy number plasmid	New England Biolabs	carbenicillin, kanamycin
pCR2.1	Topo cloning vector, high copy number, pUC ori	Invitrogen-Life Technologies	carbenicillin, kanamycin
pCR_rovM	pCR2.1 derivative with *rovM* gene and native promoter region	This study	carbenicillin, kanamycin
pACrovM	pACYC177 derivative with *rovM* gene and native promoter region	This study	carbenicillin
D. *Yersinia pestis* KIM6+ strains			
**Strain**	**description**	**source**	
KIM6+ wild type (WT)	wild type strain		
*ΔrovM*	*rovM* gene *(y1629*) replaced by kanamycin gene cassette	This study	
WT (pCR2.1)	wild type strain harboring pCR2.1	This study	
**Strain**	**description**	**source**	
WT (pACYC177)	wild type KIM6+ strain harboring empty vector pACYC177	This study	
**plasmid**	**description**	**source**	
WT (pCR_rovM)	wild type KIM6+ strain harboring pCR_rovM plasmid	This study	
*ΔrovM* (pACYC177)	mutant in *rovM* gene harboring empty vector pACYC177	This study	
*ΔrovM* (pACrovM)	mutant in *rovM* gene harboring pACrovM	This study	
*ΔrovM* (pCR_rovM)	mutant in *rovM* gene harboring pCR_rovM	This study	

### Flea infections

*Y*. *pestis* KIM6+ (pACYC177), *ΔrovM* mutant (pACYC177) and *ΔrovM* (pACrovM) strains used for flea infections were grown in Brain Heart Infusion (BHI) broth overnight twice in succession, first at 28°C and then at 37°C without aeration. Bacterial cultures were centrifuged, and the bacterial pellet was resuspended in 0.5 ml PBS and added to 5 ml of fresh heparinized mouse blood at a concentration of approximately 1X10^9^ cells/mL. *Xenopsylla cheopis* fleas were then allowed to feed on the infected blood using a previously described artificial feeding chamber [21, 22]. Fleas that took a blood meal were maintained at 21°C and 75% relative humidity, fed twice weekly on uninfected mice and monitored for proventricular blockage for 28 days as previously described [21]. The infection rate was determined by cfu count of the bacterial load in samples of 20 infected fleas collected immediately after the infectious blood meal, 7 days and 28 days post-infection [21, 23].

Co-infections were performed similarly to single infections as previously reported [19, 24]. The fleas were allowed to feed on blood containing an approximate 1:1 ratio of either the Δ*rovM* mutant or the Δ*rovM* (pACrovM) with the wild-type strain. Bacterial loads of 16–20 infected fleas were determined at 0 and 28 days post-infection, by plating on Yersinia selective agar base (Thermo) supplemented with 1μg/μL irgasan only (YSAB-irg) to determine total cfu*Y*. *pestis* per flea. Simultaneously, plating was carried out on YSAB-irg plus 50μg/mL kanamycin, or 100μg/mL carbenicillin, to select for the Δ*rovM* mutant or Δ*rovM* (pACrovM) respectively. Two independent co-infection assays were performed.

### Ethics statement

This study was carried out in strict accordance with the recommendations in the Guide for the Care and Use of Laboratory Animals of the National Institutes of Health. The protocol (#04001) was approved by the Committee on the Ethics of Animal Experiments of Washington State University.

### RT-qPCR

RNA was isolated from three independent exponential phase cultures and from fleas using the RNeasy RNA isolation kit (Qiagen) and was treated with rDnase I (Ambion) to remove contaminating genomic DNA. For RNA isolation from fleas, triplicate independent pools of ~35 flea guts from fleas infected with the Δ*rovM* or wild-type strain was processed two weeks post-infection as previously described [1]. RNA quality was assessed using an Agilent Bioanalyzer 2100 (Agilent Technologies) and only samples with RIN values of ≥8 and A_260_/A_280_ ratios of ~2.0 were used. To confirm that the samples were free of genomic DNA real time PCR of RNA was performed. cDNA was synthesized from 2–5 μgs total RNA using the Superscript III reverse transcriptase (Invitrogen) as per manufacturer’s instructions. Each 25μL quantitative PCR reaction was carried out using 20ngs of cDNA per sample, in triplicate and the Taqman Universal PCR Master Mix (Life Technologies) on an ABI Prism 7900 sequence detection system (Applied Biosystems) using the following conditions: 95°C for 10 min, followed by 40 cycles of 95°C for 15s and 60°C for 1min. The Taqman primer and probes sets were designed using Primer Express version 2.0 software (Life Technologies) and are listed in Table 1. Primers and probes sets for all three genes, were used at 500nM and 250nM final concentration, respectively. For each primer-probe set assay, a standard curve was prepared using known concentrations of *Y*. *pestis* KIM6+ genomic DNA. Standard curves were used to transform C_T_ values into relative DNA quantity. The quantity of cDNA for each experimental gene was normalized relative to the quantity of the reference gene *crr* (y1485), whose expression is not affected by *in vivo* or *in vitro* growth conditions [1, 25, 26]. The S1 Dataset contains raw data from the RT-qPCR using SMM medium conditions for which RT-qPCR has not previously been performed using the *crr* normalization control gene. Each assay was performed in triplicate for the three independent biological samples. To calculate fold change we used a ratio of the normalized values calculated for the *gene of interest*/*crr* between two strains e.g fold change in *gene of interest* transcript between strain A and strain B will be calculated by deriving a ratio from (*gene of interest* /*crr*)_strain A_/ (*gene of interest* /*crr*) _strain B_.

### Statistical analysis

The GraphPad Prism 5 software was used to statistically analyze the data. A One Way ANOVA with a Tukey’s multiple comparison post-test was used to determine any significant differences in biofilm formation, growth rates and transcript levels. For flea co-infection assays, a Student’s t-test was used to determine significant difference between the mean percentage infection per strain at days 0 and 28, post-infection. Linear regression analysis of the exponential phase of growth was performed to determine the growth rate (μ) of each strain.

## Results

### *Y*. *pestis* strains harboring multiple plasmid-encoded copies of *rovM* show differential biofilm formation in response to nutritional cues

Homologs of RovM in *E*. *coli* (LrhA) and *Erwinia chrysanthemi* (PecT) have been implicated in the regulation of genes required for aggregation and biofilm production [3, 5]. Over-expression associated with the *rovM* homolog cloned on a multicopy plasmid have been implicated in aberrant phenotypes, while the wild type and mutant strains exhibit equivalent phenotypes e.g over-expression of *lrhA* from a low copy number plasmid resulted in loss of motility in *E*. *coli* while the *ΔlrhA* mutant and wild type remained motile. [3].

**Fig 1:**
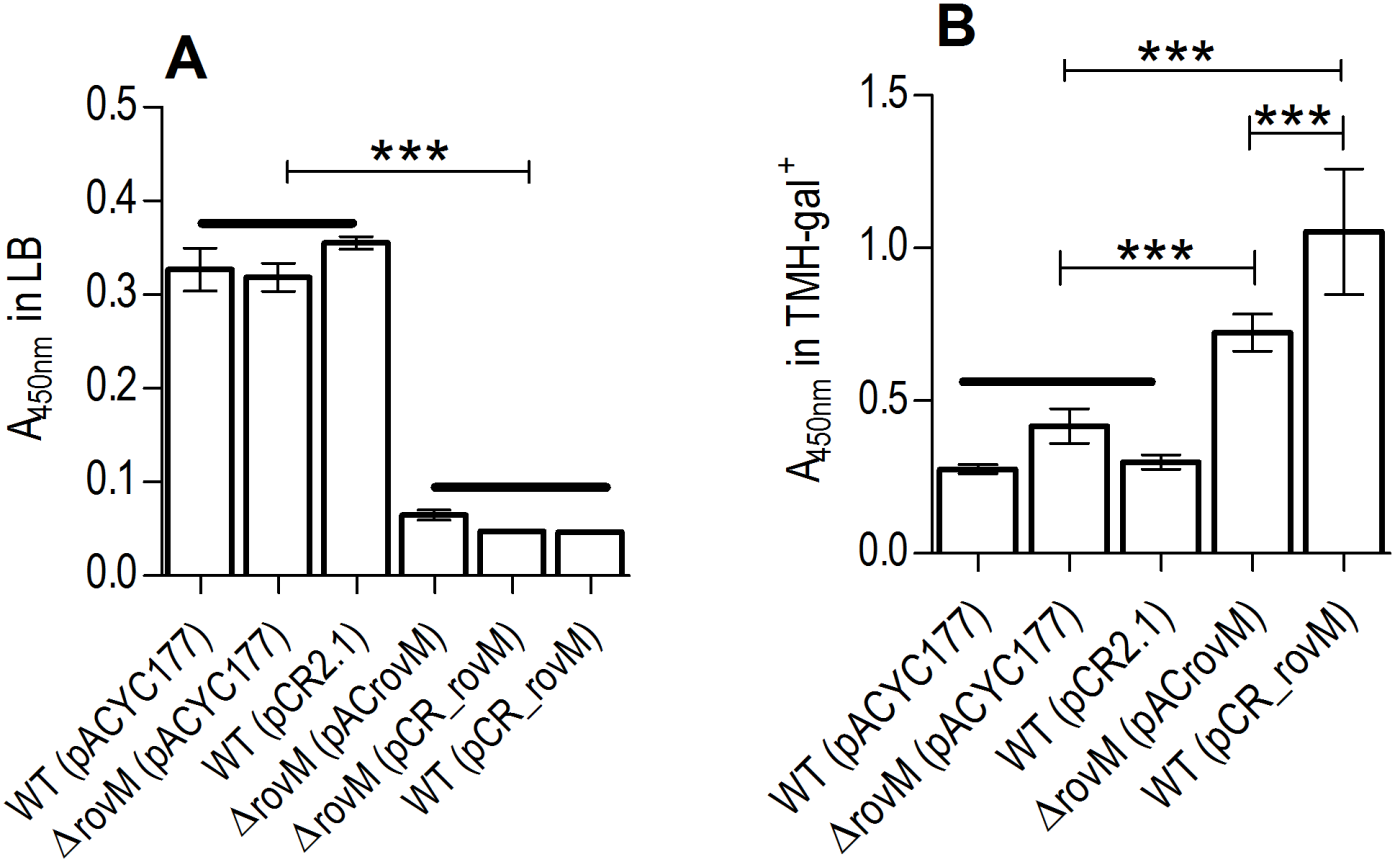
*Y*. *pestis* strains with multiple plasmid-encoded copies of *rovM* show differential ability to form biofilm in varying nutritional conditions. Biofilm formation of *Y*. *pestis* KIM6*+* strains in LB medium supplemented with 4mM MgCl_2_ and 4mM CaCl_2_ (A) and TMH-gal^+^ medium (B). Error bars are the mean±SD of four independent biological replicates. A One Way ANOVA combined with a Tukey’s test was used to test significant differences in the biofilm formation between strains. The overall P-value for the ANOVA was P<0.0001. *** represents P<0.001 and indicates a significant difference in biofilm formed between the strains. Horizontal lines mark a groups of bars that are being compared.

**Fig 2:**
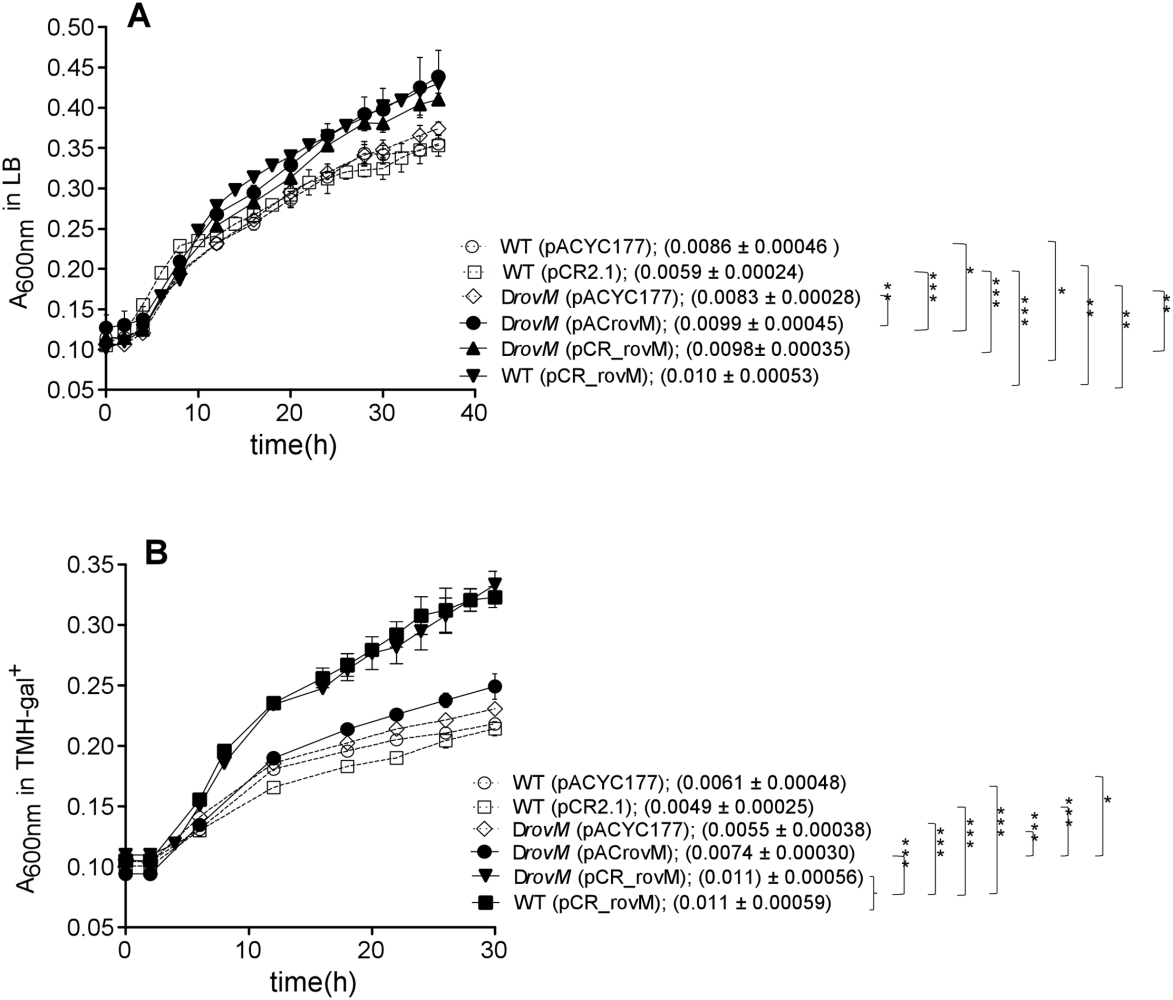
*Y*. *pestis* strains with multiple plasmid-encoded copies of *rovM* grow at a faster rate. Growth of *Y*. *pestis* strains in LB medium (A) and TMH supplemented with 0.2% galactose (B). Growth curves are the mean±SD of four independent biological replicates. The mean mean±SD of the growth rate (μ) is given in parentheses. All closed symbol growth curves represent strains containing multiple copies of *rovM*. A One Way ANOVA combined with a Tukey’s test was used to test significant differences in the growth rate between strains. The overall P-value for the ANOVA was P<0.0001. Significant differences in μ between strains are indicated by *** for P<0.001, ** for P<0.01 and * for P<0.05. Curly brackets group together strains that do not have significant differences. Capped bars indicate strains that are being compared.

Therefore the ability of a Δ*rovM* mutant strain to successfully produce a biofilm on polystyrene microtiter plates in comparison to the wild type, and wild type and Δ*rovM* mutant strains transformed with a plasmid harboring the *rovM* gene (wild type (pCR_rovM), *ΔrovM* (pACrovM), and *ΔrovM* (pCR_rovM)) was assessed (Fig 1). The Δ*rovM* mutant, produced similar quantities of adherent biofilm relative to the wild type strains containing either high or low copy number empty plasmids, in LB (4mM MgCl_2_, 4mM CaCl_2_) medium (Fig 1A). Interestingly, both wild type and Δ*rovM* strains, transformed with pCR_rovM or pACrovM, such that they contained multiple copies of the *rovM* gene, appeared to produce equally reduced biofilm, but this was due to less adherent biofilm easily washing away. Growth analyses in LB medium and TMH medium (supplemented with 0.2% galactose), demonstrated that wild type (pCR_rovM), *ΔrovM* (pCR_rovM) and *ΔrovM* (pACrovM) grew at significantly faster rates than wild type and the *rovM* mutant (Fig 2).

When cultured in TMH-gal^+^ medium, biofilm was produced in larger amounts by the wild type (pCR_rovM) and Δ*rovM* (pACrovM) strains relative to the comparable biofilm formed by the wild type and Δ*rovM* mutant strains containing empty plasmids (Fig 2B). Dissimilarly, less adherent biofilm was not observed to be the reason for this difference in TMH-gal^+^ medium.

### A Δ*rovM* mutant has a competitive fitness disadvantage in the flea

The RovM homologs LrhA and HexA of the insect pathogens, *P*. *temperata* and *X*. *nematophila*, regulate expression of virulence factors, immuno-suppression and metabolic usage during infection of their respective insect hosts [6, 8]. We observed differential biofilm formation in *rovM* over-expressing strains in different nutritional environments. We therefore predicted that if *Y*. *pestis* RovM maintained similar roles during flea infection, then its absence may impact growth, survival, and/or biofilm-mediated gut blockage.

**Fig 3:**
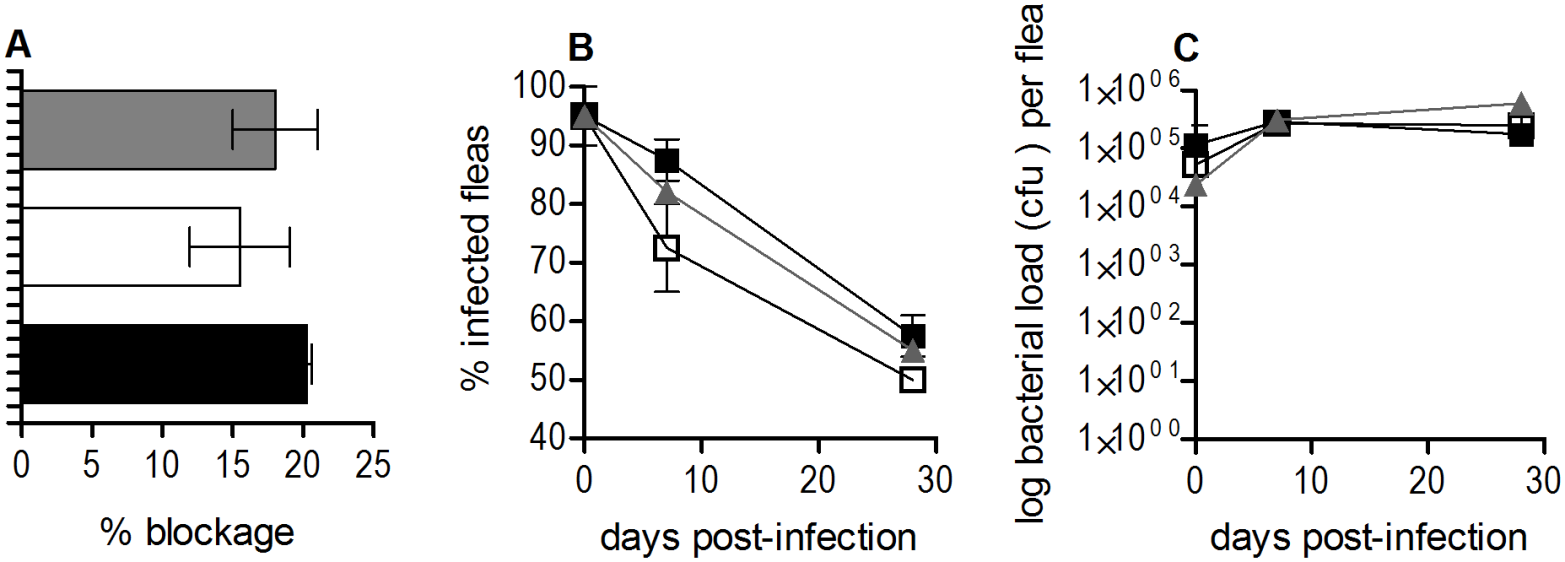
RovM does not contribute to biofilm blockage, growth and survival of *Y*. *pestis* in the flea gut. Flea infection of the *Y*. *pestis* KIM6+ WT (grey), *Y*. *pestis* KIM6+ Δ*rovM* (pACYC177) mutant (black) and corresponding *Y*. *pestis* KIM6+ Δ*rovM* (pACrovM) complemented mutant strain (unfilled). The percentage of fleas that develop blockage after infection (A), percentage of infected fleas (B) and average bacterial load (cfu/flea) (C) are not different between the Δ*rovM* (pACYC177) mutant, wild type or complemented Δ*rovM* (pACrovM) strains for the 2–3 independent flea infection studies presented. An average of 20 fleas per strain at each time point represents data for cfu/flea and percentage of infected fleas for the studies. Error bars are the mean±SD of 2–3 independent biological replicates.

Therefore, the ability of a Δ*rovM* mutant strain of *Y*. *pestis* to successfully infect and produce biofilm blockage in fleas was assessed (Fig 3). During flea infection the wild type, Δ*rovM* mutant and complemented mutant Δ*rovM* (pACrovM) strain displayed comparable biofilm blockage (Fig 3A), infection rates (Fig 3B), average bacterial loads per flea (Fig 3C), and flea mortality (data not shown).

**Fig 4:**
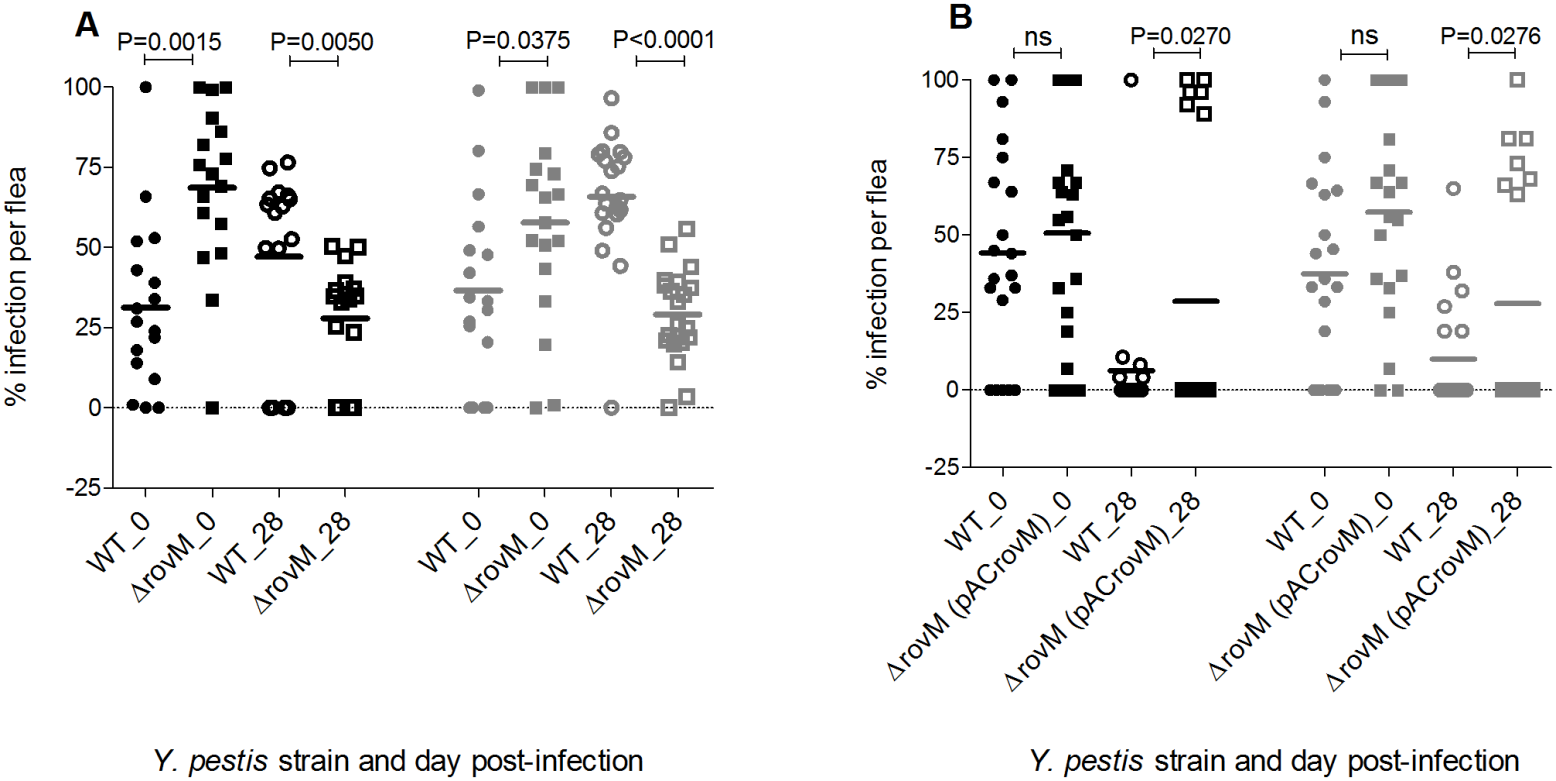
Deletion of *rovM* in *Y*. *pestis* confers a competitive fitness defect while the complemented mutant, *rovM* (pACrovM) has a competitive fitness advantage, during flea infection. Percentage infection per flea at 0 and 28 day post-infection, with (A) a *rovM* mutant and wild type strain or, (B) *rovM* (pACrovM) and the wild type strain. The mean of the percentage infection for 16–20 fleas is indicated by the horizontal bar. A student’s t-test was used to determine the significant differences between the percentage infections of each strain in fleas. The P values are given. Black symbols represent the first infection and grey symbols the second infection.

If *Y*. *pestis* is deleted of a gene whose product is important for adaptation in the flea gut, a fitness defect could result from its competitive interactions, leading to a less transmissible infection. Therefore to investigate if any potential liability was imposed by a mutation of RovM, a competitive co-infection of this mutant with its isogenic wild type strains was performed. A cohort of fleas was co-infected in a 1:1 ratio with the wild type and Δ*rovM* mutant or Δ*rovM* (pACrovM) strain (Fig 4). During co-infection with the wild type a significant decrease in the Δ*rovM* mutant flea infection load was observed at day 28 post-infection, despite its apparent significantly increased infection load at day 0 post-infection. In converse, during a co-infection with the wild type strain, the Δ*rovM* pACrovM strain showed a significantly increased infection load at 28 days co-infection.

### RovM regulates growth in response to nutrients

**Fig 5:**
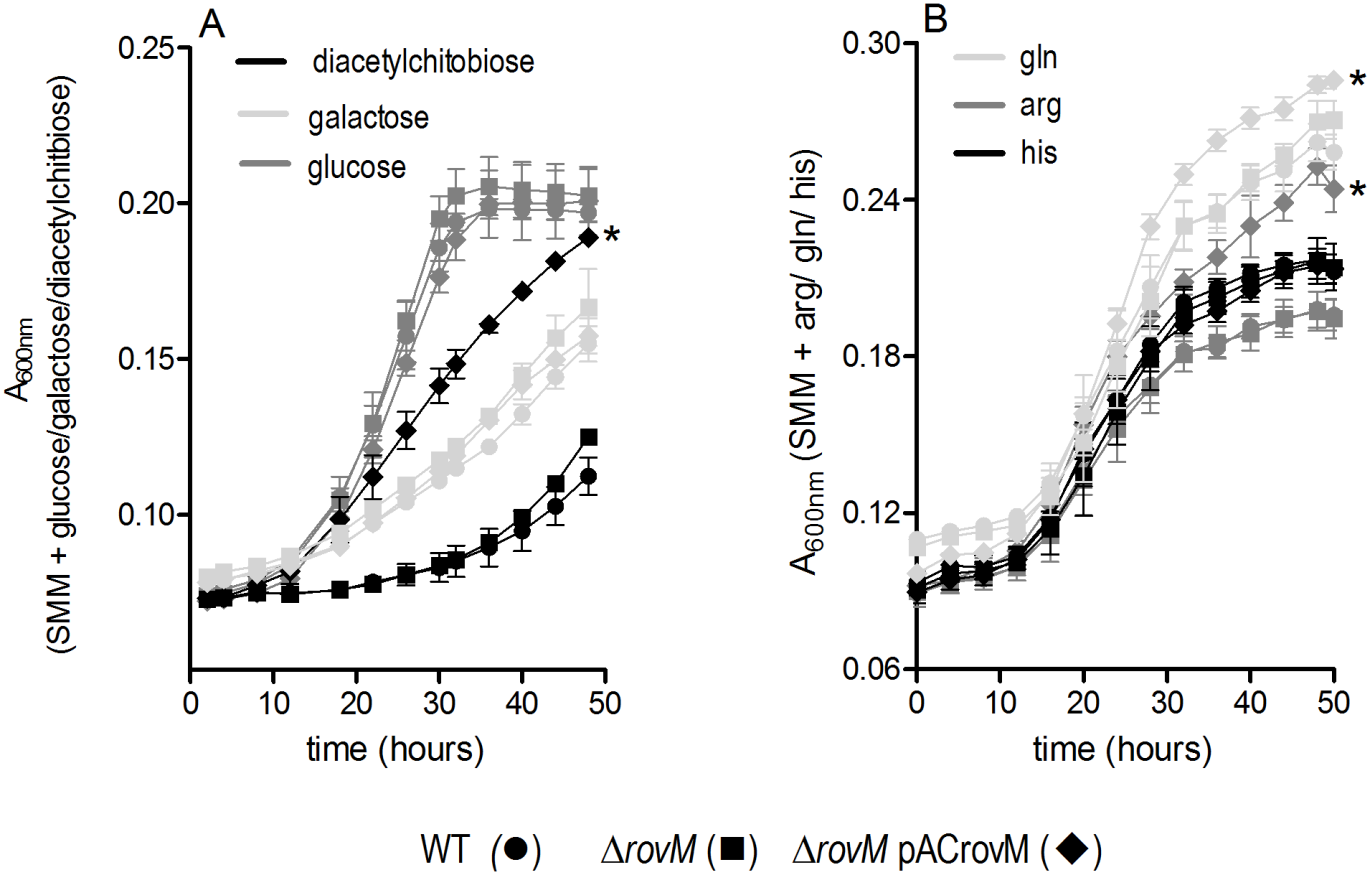
RovM senses nutritional cues. Growth of WT (pACYC177) (●), Δ*rovM* (pACYC177) mutant (■) and Δ*rovM* (pACrovM) complemented mutant (♦) strains in Sebbane minimal medium (SMM) supplemented with 0.2% (A) glucose (dark grey), galactose (light grey), or diacteylchitobiose (black) as additional carbon sources or, (B) amino acids, glutamine (light grey), arginine (dark grey) and histidine (black). Error bars indicate the mean±SD of three independent experiments. To calculate the growth rate linear regression analysis was performed on the exponential phase time points of growth between 18–32 hours for (A) and 16–32 hours for (B). The *ΔrovM* (pACrovM) complemented strain grew at a significantly higher growth rate (*, P<0.001) in SMM supplemented with diacetylchitobiose, glutamine and arginine compared to the Δ*rovM* mutant and wild type strains. WT = wild type.

LrhA mutants of *X*. *nematophila* exhibit metabolic defects that affect its ability to infect its insect host, foretelling that this regulator senses nutritional composition of its host environment [27]. To determine if *Y*. *pestis* RovM is similarly involved in metabolic processes, growth analysis of wild type, *ΔrovM* mutant and complemented mutant *ΔrovM* (pACrovM) in a very minimal medium, SMM, was performed following supplementation with sugars and amino acids that are not normally contained within SMM. Supplementing the media with glucose and galactose, resulted in no observable difference in growth rate among the strains (Fig 5A), however, diacetylchitobiose supplementation resulted in significantly higher growth rate for the Δ*rovM* (pACrovM) strain as compared to the *rovM* mutant and the wild type strains. Maltose and arabinose supplementation revealed no growth rate differences between strains (data not shown).

Predominant usage of the L-glutamate group of amino acids (pro, his, arg, gln) is predicted to support *Y*. *pestis* growth in the flea gut, therefore his, arg, and gln were tested for their ability to support improved growth of *Y*. *pestis* in SMM (Fig 5B). Interestingly, while the Δ*rovM* mutant and wild type grew similarly in gln and arg, the Δ*rovM* (pACrovM) strain appeared to have a significantly enhanced growth rate when supplemented with these amino acids. Histidine addition however supported equivalent growth rates for all the strains.

### Specific nutritional environments direct induction of *rovM* and RovM-associated repression of *rovA*

**Fig 6:**
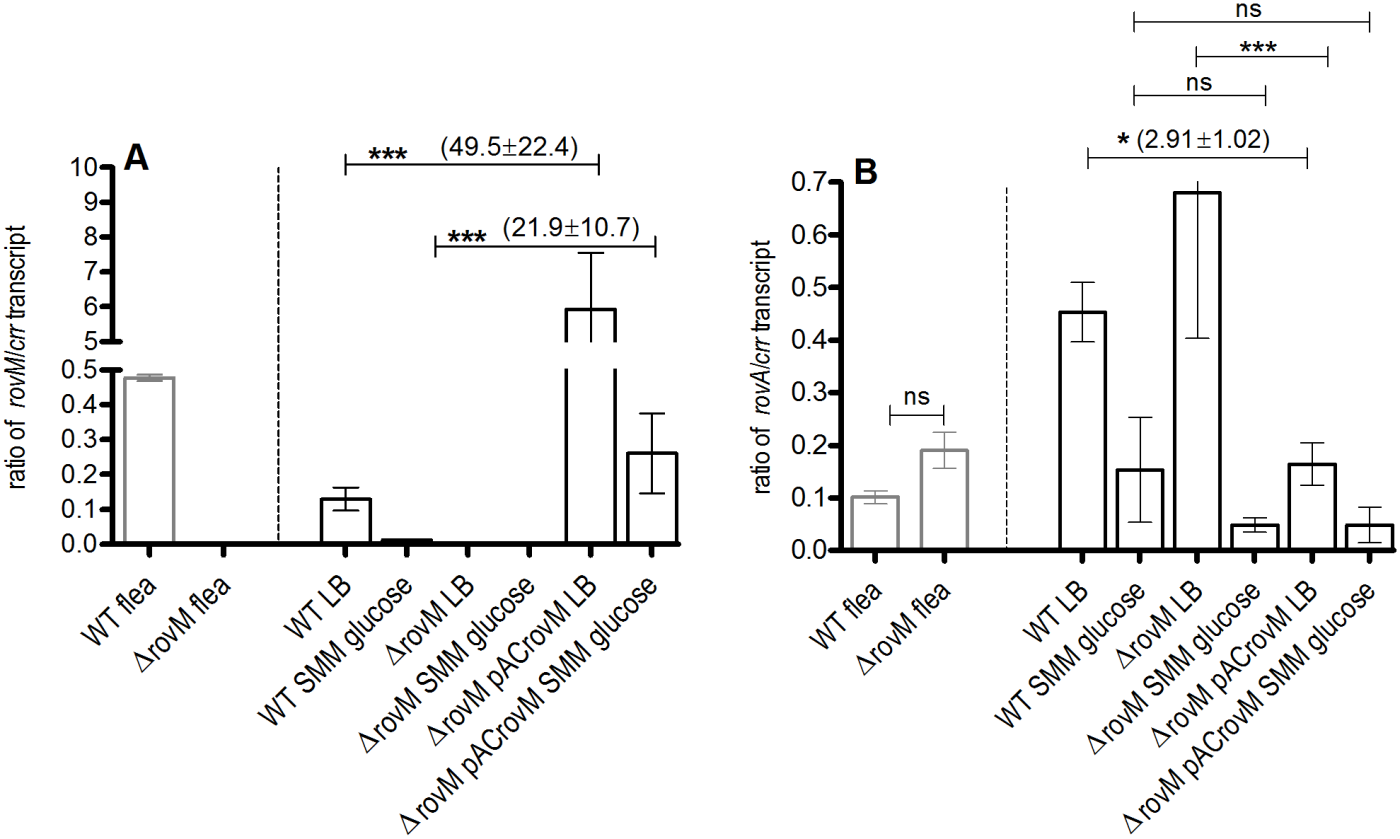
Transcription of *rovA* and *rovM* in distinct nutritive media. Quantitative transcript levels of (A) *rovM* and (B) *rovA* genes from exponential phase planktonic cultures of WT (pACYC177), Δ*rovM* (pACYC177) mutant and Δ*rovM* (pACrovM) complemented mutant strains in fleas (light grey bars), LB, and Sebbane minimal medium supplemented with 0.2% glucose. Error bars indicate the mean± SD of three independent experiments. A One Way ANOVA combined with a Tukey’s test was used to test significant differences in transcript levels between strains. The overall P-value for (A) and (B) is P<0.0001. Significant differences in gene transcript levels between stains is indicated by *for P<0.05 and *** for P<0.001. Average fold increased expression±SD of *rovM* and *rovA* is indicated in parentheses. WT = wild type, ns = not significant.

In *Y*. *pseudotuberculosis*, the RovM regulator, represses *rovA* specifically in a minimal nutritional environment [10], and in *Y*. *pestis*, *rovM* is highly induced, while *rovA* is highly repressed in fleas [1]. We hypothesized that in *Y*. *pestis* similar and specific nutritional environments would cause repression of *rovA* by RovM, and that this included the flea gut. However, gene expression analysis of flea infections of a Δ*rovM* mutant strain, showed no significant difference in *rovA* expression between the wild type and Δ*rovM* mutant (Fig 6A and 6B). Following this finding, *in vitro* transcriptional levels of *Y*. *pestis rovA* and *rovM* in response to differing nutritional environments was also determined. This was performed to establish the inducing environments of *rovM* and *rovA*, and whether a reciprocal modulatory association between the 2 regulators in certain nutritional environments does indeed occur. In *Y*. *pestis*, the *rovM* gene had lower transcriptional levels in minimal SMM media versus rich LB in the wild type strain (Fig 6A). The *rovA* transcript levels were ~3-fold higher than *rovM* and no difference in *rovA* levels was present between the wild type and Δ*rovM* mutant in both media (Fig 6B).

However, the Δ*rovM* (pACrovM) strain exhibited ~49 and ~22-fold increases in *rovM* gene expression in LB and SMM glucose, respectively, relative to the wild type growing in the same medium. Simultaneously, expression of *rovA* was repressed ~ 3-fold in the *rovM* over-expressing strain, Δ*rovM* (pACrovM) in LB medium but no difference in expression of *rovA* was noted in SMM glucose. The levels of *rovA* was however not affected by *rovM* in minimal SMM medium supplemented with glucose.

### Arginine is sensed by RovM to regulate growth fitness and biofilm production

**Fig 7:**
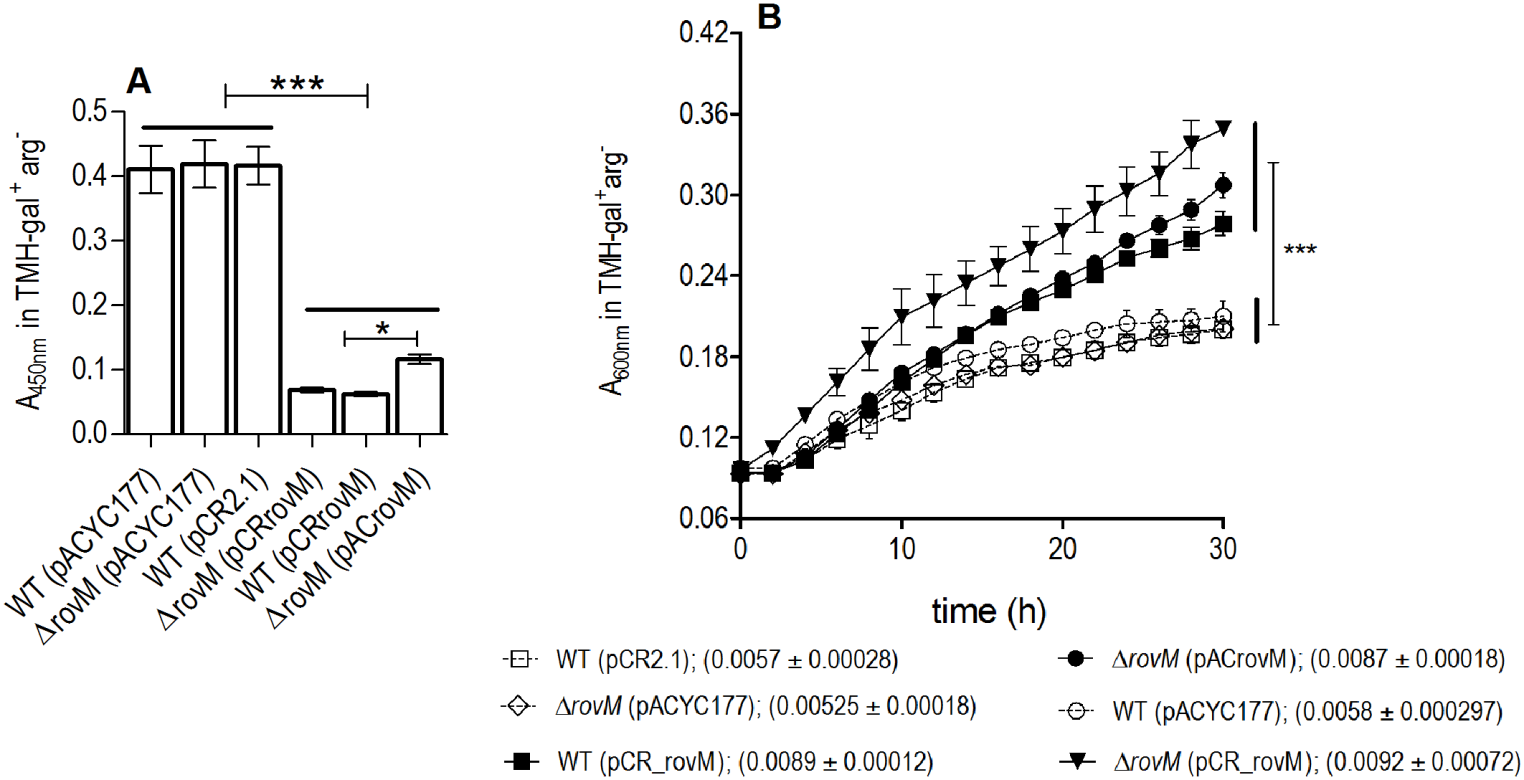
Arginine is sensed by RovM to control biofilm production. (A) Biofilm formation of *Y*. *pestis* KIM6+ strains in TMH-gal^+^arg^-^. Error bars are the mean±SD of four independent biological replicates. A One Way ANOVA combined with a Tukey’s test was used to test significant differences in biofilm formation between strains. The overall P-value for the ANOVA was P<0.0001. Significant differences in biofilm formation are indicated by * for P<0.05 and *** for P<0.001. (B) Growth of *Y*. *pestis* strains in TMH-gal^+^arg^-^. Error bars are the mean±SD of four independent biological replicates. The mean mean±SD of the growth rate (μ) is given in parentheses. All closed symbol growth curves represent strains containing multiple copies of *rovM*. A One Way ANOVA combined with a Tukey’s test was used to test significant differences in the growth rate between strains. The overall P-value was P<0.0001. Significant difference in growth rates is indicated by *** for a P-value of 0.0001. Horizontal lines group a set of bars together. Capped lines mark the two bars (or groups of bars) that are being compared.

**Fig 8:**
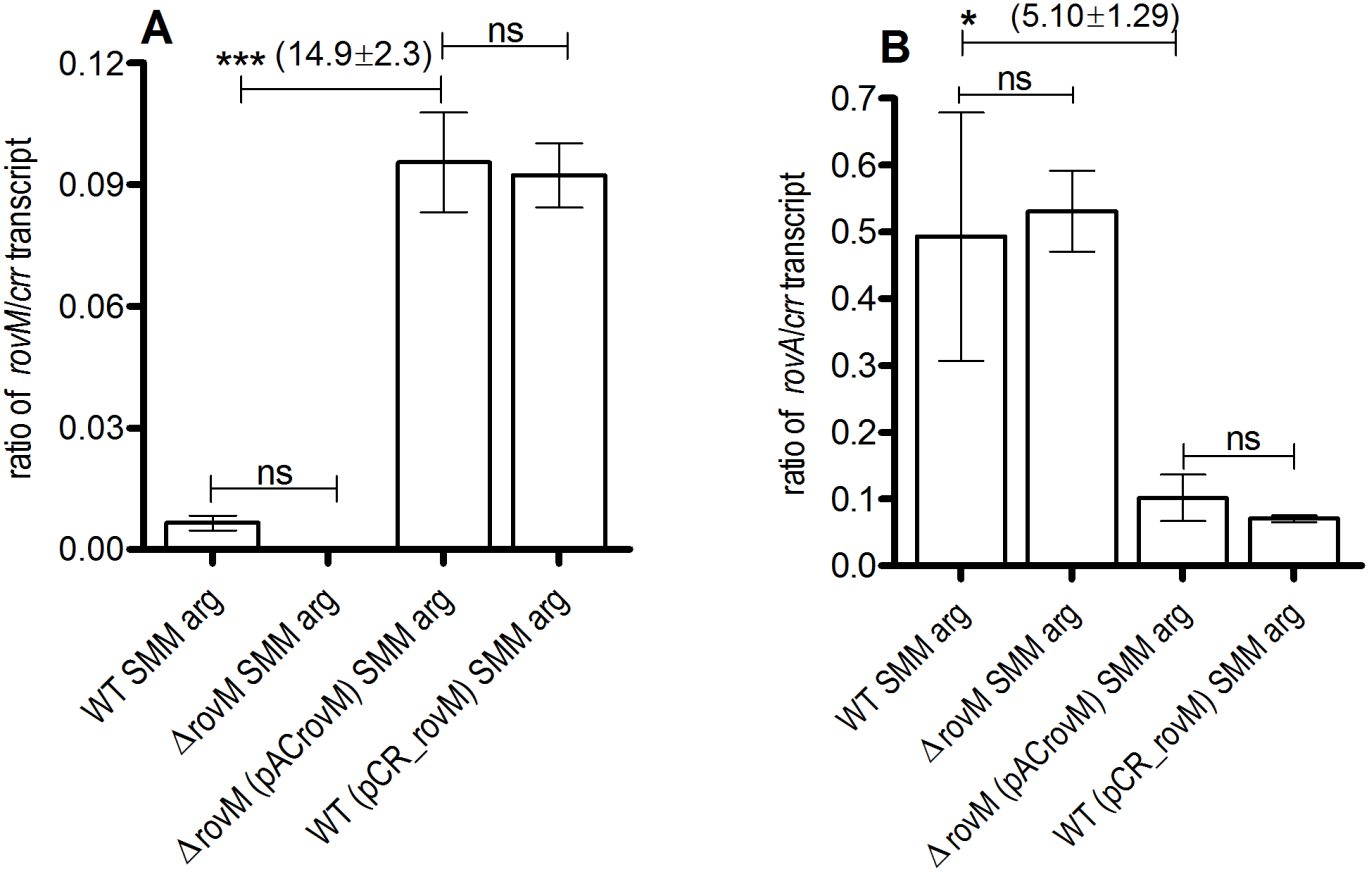
RT-qPCR of *rovM* and *rovA* in minimal medium containing arginine. Quantitative transcript levels of (A) *rovM* and (B) *rovA* genes from exponential phase planktonic cultures of wild type (pACYC177), Δ*rovM* (pACYC177) mutant, wild type (pCRrovM) and Δ*rovM* (pACrovM) complemented mutant in Sebbane minimal medium supplemented with arginine. Error bars represent the mean±SD of at least 3 independent replicates. A One Way ANOVA combined with a Tukey’s post-test was used to test significant differences in transcript level. The overall P-value for the ANOVAs was 0.0003 for (A) and 0.0173 for (B). Significant differences in gene transcript levels between strains is indicated by *for P<0.05 and *** for P<0.001 Average fold increase in expression±SD of *rovM* and *rovA* is indicated in parentheses. WT = wild type, ns = not significant.

Supplementation of SMM with arg as the novel nitrogen source led to increased growth rate of the Δ*rovM* (pACrovM) strain (Fig 5B). Arg is predicted to be catabolized by *Y*. *pestis* in the flea gut, therefore the ability of the wild type, Δ*rovM* mutant and complemented mutant, Δ*rovM* (pACrovM) to respond to arg in the context of biofilm production was tested (Fig 7A, S1 Dataset). Lack of arg in TMH-gal^+^arg^-^ medium, resulted in significant decreases in biofilm formation in strains with multiple copies of *rovM*, Δ*rovM* (pACrovM) and wild type (pCR_rovM) (Fig 7A). However, the wild type and Δ*rovM* mutant strains were able to make comparable amounts of biofilm independent of the presence of arg in TMH medium (Figs 1B and 7A). The growth rate of strains harboring multiple copies of *rovM* was higher than the wildtype and Δ*rovM* mutant in TMH-gal^+^arg^-^ medium (Fig 7B), similar to growth in TMH-gal^+^ (Fig 2B). To assess if any differences in *rovM* transcription occurs in the presence of arg, the *rovM* expression levels in SMM medium supplemented with arg was determined. Arg addition did not result in induction of *rovM* expression as noted by the similar expression levels of *rovM* in the wild type strain in SMM plus glucose and SMM plus arg (Figs 6A and 8). However, in SMM arg, over-expression of *rovM* occurs in the Δ*rovM* (pACrovM) and wild type (pCR_rovM) strains with concomitant *rovA* repression in keeping with what occurs in LB medium.

## Discussion

To understand which *Y*. *pestis* genes were essential for flea gut colonization and adaptation to produce a biofilm blockage-mediated transmissible infection we defined the unique transcriptome of *Y*. *pestis* in blocked fleas relative to LB *in vitro* growth conditions previously [1]. Genes that were uniquely highly induced in the flea gut environment were assumed to be prioritized for flea adaptation and transmission, urging a functional understanding of their roles during *Y*. *pestis* flea infection. This study aimed to characterize the functional role of one such gene, *rovM* that encodes a LTTR.

Our data from this study suggests that *rovM* may be induced in *Y*. *pestis* during flea infection by available carbons and nitrogen sources or perhaps other environmental factors. The competitive fitness defect exhibited by the *rovM* mutant during co-infection with the wild type suggests that lack of RovM leads to a deficiency at acquiring available nutrients in the flea gut. We previously showed that *Y*. *pestis* has a distinctive metabolism in the flea gut which is characterized mainly by predominant usage of peptides and amino acids along with lipid and some pentose carbohydrate utilization [1]. Specifically induction of genes involved in the uptake and catabolism of diacetylchitobiose (*chbBC*, *chbF*), arg (*artQ*), and gln (*glnQPH*), occurred alongside induced *rovM* expression in fleas [1]. In this study we demonstrated that these three metabolites support a growth fitness advantage of *Y*. *pestis* overexpressing *rovM in vitro*. Alternately, catabolism of hexose sugars is not thought to be significant in the flea gut [1, 28] and we observe that *rovM* over-expression does not impact growth fitness of *Y*. *pestis* in the presence of the hexose sugars, glucose, galactose or maltose. The flea blood meal and its catabolic by-products produced from digestion by the flea and its inherent gut microbiota serve as the nutritional source for *Y*. *pestis* survival and growth in the flea gut [28–31]. Therefore *Y*. *pestis* may not only be in competition with the flea and its resident microbiota for the blood meal, but it may need to be capable of utilizing by-products of bloodmeal digestion produced by the flea and its microbiota. The transcriptional regulator, RovM likely directs more efficient utilization of some available nutrients in the flea gut to support *Y*. *pestis* adaptation to this environment. Certainly the improved fitness during co-infection with the wild type displayed by the, Δ*rovM* pACrovM strain that over-expresses *rovM*, supports this notion. This ability to respond to nutritional cues is a function that has been attributed to homologs of RovM. For example, LrhA in *E*. *coli*, negatively regulates galactose and aspartate chemoreceptors thereby controlling bacterial motility in response to acquisition of these nutrients [3]; *Y*. *pseudotuberculosis* RovM is induced when cultured in minimal medium, [10]; LrhA of *X*. *nematophila* supports propagation of this insect pathogen in a minimal medium containing specific amino acids [27], whereas an Δ*lrhA* mutant shows no growth differences relative to the wild type strain in LB medium. Richards and Goodrich-Blair [27] suggest that metabolites can act as transition signals that might enable pathogen switching from virulence to metabolic activities, or that consumption of specific nutrients during infection may serve as a signal to express genes required for transmission.

Our results indicate that augmented expression of *rovM* in a complex rich LB medium, leads to less adherent biofilm formation, whereas in the nutritionally defined TMH medium containing arg, more biofilm is produced. Furthermore, *rovM* over-expression correlates with a faster growth rate in the presence of arg in minimal medium. Together this implies that RovM influences biofilm formation via integration of nutritional cues and that arg may be central to this signaling. This is in line with the idea that metabolites may act as transition signals. Formation of biofilm in *Y*. *pestis* occurs in the flea host only, and is important for biofilm-mediated transmission of plague and to maintain persistent colonization of the flea gut [21, 22]. The *hms* group of genes, comprising the *hmsHFRS* gene locus and single genes, *hmsT* and *hmsP* are responsible for synthesis and regulation of the extracellular polysaccharide matrix (ECM) that is fundamental to biofilm formation [22, 32]. The ECM facilitates biofilm-mediated transmission and persistence in the flea gut [21, 22]. Polyamines are also necessary for formation of *Y*. *pestis* biofilm through their modulation of HmsR and HmsT protein translation. Interestingly arg is required as a precursor in the first step of polyamine biosynthesis where it is converted by the arginine decarboxylase to agmatine.

Only elevated transcript levels of *rovM*, occurring through *in trans* over-expression of the *rovM* gene (e.g. pACrovM and pCR_rovM here) in Δ*rovM* mutant and wild type bacteria resulted in notable alterations in biofilm, flea infection and growth fitness. This is consistent with studies focused on *Y*. *pseudotuberculosis* [10] and *E*. *coli* [3] RovM homologs wherein only elevated levels of RovM could be correlated with notably distinct aberrant phenotypes when no difference occurred in the wild type and RovM-homolog mutant strains. For instance elevated levels of RovM in *Y*. *pseudotuberculosis* resulted in hypermotility and virulence attenuation in mice while there were no apparent differences between the wild type and Δ*rovM* mutant strains [10]. Furthermore, in the case of plasmid-mediated over-expression of *lrhA* in *E*. *coli*, motility was decreased when compared to motility in an *lrhA* mutant carrying an empty plasmid [3]. The crystallographic structure of *Y*. *pseudotuberculosis* RovM reveals a potential requirement for a metabolic inducer molecule to mediate its folding [33] into an active tetrameric unit. Formation of the tetrameric unit is thus essential for RovM regulatory function, which is consistent with the function of other LTTRs. It can then be assumed that *in trans* over-expression of *rovM* will result in more functionally active units of RovM, as compared to the wild type and Δ*rovM* which synthesize few to no transcripts of *rovM*. Another hallmark of LTTRs is autoregulation [10, 34], and explains that induced RovM synthesis leads to further induction of *rovM* expression. Between 2–15 copies of the *rovM* gene is expected to be present in the Δ*rovM* (pACrovM) strain, based on the copy number of the pACYC177 plasmid [35]. Induction of *rovM* expression in pACrovM transformed strains, may therefore lead to further induction of *rovM*, accounting for the strident differences in *rovM* expression between these strains and the wild type and Δ*rovM* strain in LB and SMM media (Fig 6).

In conditions when *rovM* is induced, or over-expressed, as observed in the pACrovM and pCR_rovM transformed strains, or naturally during flea infection, more functional RovM units are present to mediate repression of *rovA* or regulation of metabolic processes. The nutritional specificity for RovM induction and for RovM-dependent repression of *rovA* are different in *Y*. *pestis* and *Y*. *pseudotuberculosis*. The induction of *rovM* and its repression of *rovA* during growth of *Y*. *pestis* in the flea matches what occurs with *in vitro* grown *Y*. *pseudotuberculosis* in minimal medium at low temperatures [10]. However, a *Y*. *pestis* Δ*rovM* mutant sustains low *rovA* transcription while growing in the insect host, which is in contrast to *Y*. *pseudotuberculosis* RovM mediated repression of *rovA* [10]. It appears that *Y*. *pestis rovA* regulation in the flea gut and in SMM glucose is not mediated through RovM, and probably requires other regulatory factors, similar to temperature and growth phase dependent *rovA* expression in *Y*. *pseudotuberculosis* [10].

*Y*. *pestis* has diverged from *Y*. *pseudotuberculosis* to lose motility, flagella synthesis [36] and production of invasin, which are all RovM-regulated virulence factors that enhance the invasiveness of *Y*. *pseudotuberculosis*. The role of RovM in regulating motility independent of RovA [10] is a conserved regulatory function of RovM homologs across a variety of pathogenic bacterial species e.g., LrhA of the nematode pathogen, *X*. *nematophila* [8], LrhA of *E*. *coli* [3] and HexA of the phytopathogen, *E*. *carotovora* [37]. These functions are absent in *Y*. *pestis* and thus no longer constitute the *Y*. *pestis* RovM regulon. During the clonal evolution of *Y*. *pestis* from *Y*. *pseudotuberculosis*, besides gains and losses of gene function through horizontal gene transfer and inactivation [36, 38], remodeling of gene regulation and global regulatory networks [39–41] has occurred. This has likely been a contributing aspect to adaptation of *Y*. *pestis* to its ability to colonize and be transmitted by fleas.

We conclude that the RovM protein may play a role in nutritional sensing and regulating physiological processes for advantageous adaptation and colonization of *Y*. *pestis* to the flea gut. This role of RovM may be particularly significant for flea-borne transmission during natural plague cycles when competitive colonization of the flea gut is more prevalent.

## Supporting Information

S1 Dataset Raw data from RT-qPCR using SMM medium. (XLSX)
